# Rapid growth rate results in remarkably hardened breast in broilers during the middle stage of rearing: A biochemical and histopathological study

**DOI:** 10.1371/journal.pone.0193307

**Published:** 2018-02-23

**Authors:** Takeshi Kawasaki, Tomohito Iwasaki, Michi Yamada, Takashi Yoshida, Takafumi Watanabe

**Affiliations:** 1 Department of Food Science and Human Wellness, Rakuno Gakuen University, Ebetsu, Hokkaido, Japan; 2 Research Office Concerning the Health of Humans and Birds, Abashiri, Hokkaido, Japan; 3 Medical Engineering Course, Graduate School of Engineering, Kitami Institute of Technology, Kitami, Hokkaido, Japan; 4 Department of Sustainable Agriculture, Rakuno Gakuen University, Ebetsu, Hokkaido, Japan; 5 Faculty of Agriculture, Shinshu University, Kami-ina, Nagano, Japan; Gaziosmanpasa University, TURKEY

## Abstract

The high incidence of meat of impaired quality poses a serious problem in the poultry industry. In recent years, the incidence of the pectoralis major muscle that appeared pale colored, remarkably hardened, and exudative, called “wooden breast” or “woody breast” has increased in slaughter houses. In the present study, 19-day-old Ross 308 broiler chickens affected (n = 10) and unaffected (n = 10) with remarkably hardened breast were selected from a commercial broiler farm, and reared to 55 days of age under a controlled environment. Among the affected birds, 5 of 10 birds appeared exhausted with markedly suppressed weight gain and 4 of 10 birds died during the rearing period. In contrast, all unaffected birds survived and most gained weight. Four of 10 unaffected birds lost the ability of back-to-back wing contact by the late stage of rearing. The biochemical analysis of blood plasma samples of 20-day-old birds revealed that creatine kinase and L-aspartate aminotransferase values in most affected birds were higher than those in unaffected birds; however, these values in unaffected birds increased rapidly with lost wing contactability and increasing age. Postmortem examinations revealed that the mean diameter of myofibers in affected birds was smaller than that in unaffected birds. Moreover, symptoms of degenerative and regenerative muscles were observed in most birds in both groups. Among them, a decrease in, or defect of, the characteristic polygonal shape of myofibers was the most common change within the pectoralis major muscles in both groups. The present study demonstrated that broilers affected with remarkably hardened breast during the middle stage of rearing would have suppressed physical status and weight gain, or would die. It was suggested that rapid growth in broilers might be a cause of remarkably hardened breast.

## Introduction

Over the past few years, degenerative lesions on the pectoralis major muscle (PM) of fast-growing broilers, which are commonly called “wooden breast” or “woody breast” myopathy, have been reported worldwide [1]. These lesions have characteristic features on a macroscopic level including pale color, remarkable hardening, exudative discharges, and sometimes manifest varying degrees of parallel white striations or petechial hemorrhages or both over the muscle surface. Histologically, hypertrophy, a wide variation in fiber size, degenerative and regenerative changes in the myofibers, and various levels of fibrosis are observed [1,2]. The severely affected muscle tissue exhibits diffuse thickening of the interstitium with varying degrees of fibrosis separating the myofibers, with occasional infiltration by inflammatory cells [1,3,4]. The lesions result in economic loss because the affected meat is either trimmed or discarded. Nevertheless, little information is currently available on wooden breast myopathy related to the incidence of clinical symptoms and passage through the rearing period.

Radaelli et al., [5] evaluated histological and immunohistochemical changes associated with the occurrence of myopathies in the PM fibers of chickens of different ages and under different conditions and revealed myofiber degeneration of the PM in 14-day-old broilers. It was previously shown that the ability of broilers to lift their wings may be a useful screening method for identifying affected live birds, regardless of the degree of wooden breast [2]. The present study aimed to investigate temporal changes in biological findings of reared birds affected or unaffected with hardened breast, determined by lifting up wings and palpating the breast from the same broiler flocks in the middle of rearing age.

## Materials and methods

### Ethics statement

All protocols and procedures were approved by the Rakuno Gakuen University Institutional Animal Care and Use Committee (No. DH16A2) in accordance with the Act on Welfare and Management of Animals of Japanese government. All birds were observed by animal stocks technicians daily, and clinical conditions were checked by poultry veterinarian as necessary during the rearing period.

### Animals and rearing management

Ten birds with remarkably hardened breasts (affected birds, No. 1–10) and 10 birds with normal breasts (unaffected birds, No. 11–20) were obtained from a commercial broiler farm, weighed, and individually tagged. These birds were Ross308, 19-day-old, and were selected following palpation and an investigation of their ability to lift their wings [2]. The tags were tied on a wing of every birds and adjusted according to increase of the body size. The birds were fed a corn-based commercial finisher mash (Metabolic energy ≥3,300 kcal) until day 55 and were reared in a clean concrete-floored pen (1.8 × 1.2 m), wherein the floor was covered with rice chaff litter. The rearing area were under suitable air conditioned and hygiene management. The birds were allowed *ad libitum* access to feed and fresh drinking water. At 27, 34, 41, 48, and 55 days of age, the birds were individually weighed. Plasma samples were taken at 20, 29, 42, and 55 days of age for biochemical analysis, after which a physical examination was performed by gently lifting up the wings of each bird to assess the ability to achieve back-to-back wing contact and palpating the breast. Blood samples were taken with sterilized disposable 2.5 ml syringes with 23 gauge needles, and collected in heparin lithium-coated tubes, and plasma was separated by centrifugation and stored at ≤−20°C until further analysis. The assessments and necropsy were not done on one affected bird (No. 4) and 3 unaffected birds (No. 11, 13 and 18) due to the purpose of other experiments, which investigate the genetic ability of wooden breast as seed chickens.

### Biochemical analysis

The plasma samples were pretreated with 1,3-dichloro-1,1,2,2,3-pentafluoropropane (FRIGEN II; Siemens Healthcare Diagnostic, Malvern, PA, USA) and diluted with purified water as necessary. A Spotchem D system (Arkray, Kyoto, Japan) was used to examine the plasma levels of creatine kinase (CK) and L-aspartate aminotransferase (AST).

### Postmortem examination and histology

Birds that died naturally (No. 1, 5, 6, and 9) during the rearing period were placed in refrigerated storage, and a postmortem examination of each bird was performed at the earliest convenience. The surviving birds (at 55 days of age), excluding No. 4, 11, 13, and 18, underwent immediate postmortem examination after euthanasia was performed by exsanguination under 20–30 mg/kg sodium pentobarbital anesthesia. Sodium pentobarbital was injected to radial vein with sterilized disposable 2.5 ml syringes with 23 gauge needles. Following postmortem examination, the length and width of the breast were measured with a ruler, and calculated breast-per-length ratio. After that, the PM, supracoracoideus muscle (SM), liver and kidney were removed and fixed in 10% formalin. These muscle samples were collected from one-third of the anterior region of each bird. After processing the samples for routine embedding in paraffin wax, sections of 5 μm thickness were cut and stained with hematoxylin and eosin. Azan, periodic acid–Schiff, or phosphotungstic acid hematoxylin staining were performed as necessary. The shortest diameters of 100 myofibers were randomly measured on the transverse sections of PM and SM of the affected birds (No. 2, 3, 7, 8, and 10) and unaffected birds (No. 12, 14, 15, 16, 17, 19, and 20). For the measurement with DP20-5 digital camera equipped with measurement software (Olympus, Tokyo, Japan) connected to BX41 optical microscope (Olympus, Tokyo, Japan), the microscopic field of vision was randomly determined, 10 fibers around the arbitrary fiber were measured, and was repeated 10 times per sample. The measurement was evaluated accuracy with carried out twice. Assessment with major histological findings of the muscles were performed with double blinded approach.

### Statistical analysis

The breast-per-length ratio and mean ± standard deviation of myofiber diameter were calculated each of birds respectively. In this study, statistical analysis was not performed in the breast-per-length ratio and myofiber diameter due to small number of the autopsied birds. The mean of body weight, CK and AST of birds in the affected or the unaffected group or the groups that were able and unable to achieve back-to-back wing contact at 55 days of age were calculated as mean ± standard deviation of the UNIVARIATE procedure. Statistical differences in the body weight, CK, and AST between the groups of affected and unaffected birds were analyzed using non-parametric Mann-Whitney (Wilcoxon Rank-Sum) Tests of the NPAR1WAY procedure. These data processing, data management, and statistical analysis were performed using in SAS (SAS University Edition, SAS Institute Inc., Cary, NC).

## Results

### Physical status

**Fig 1:**
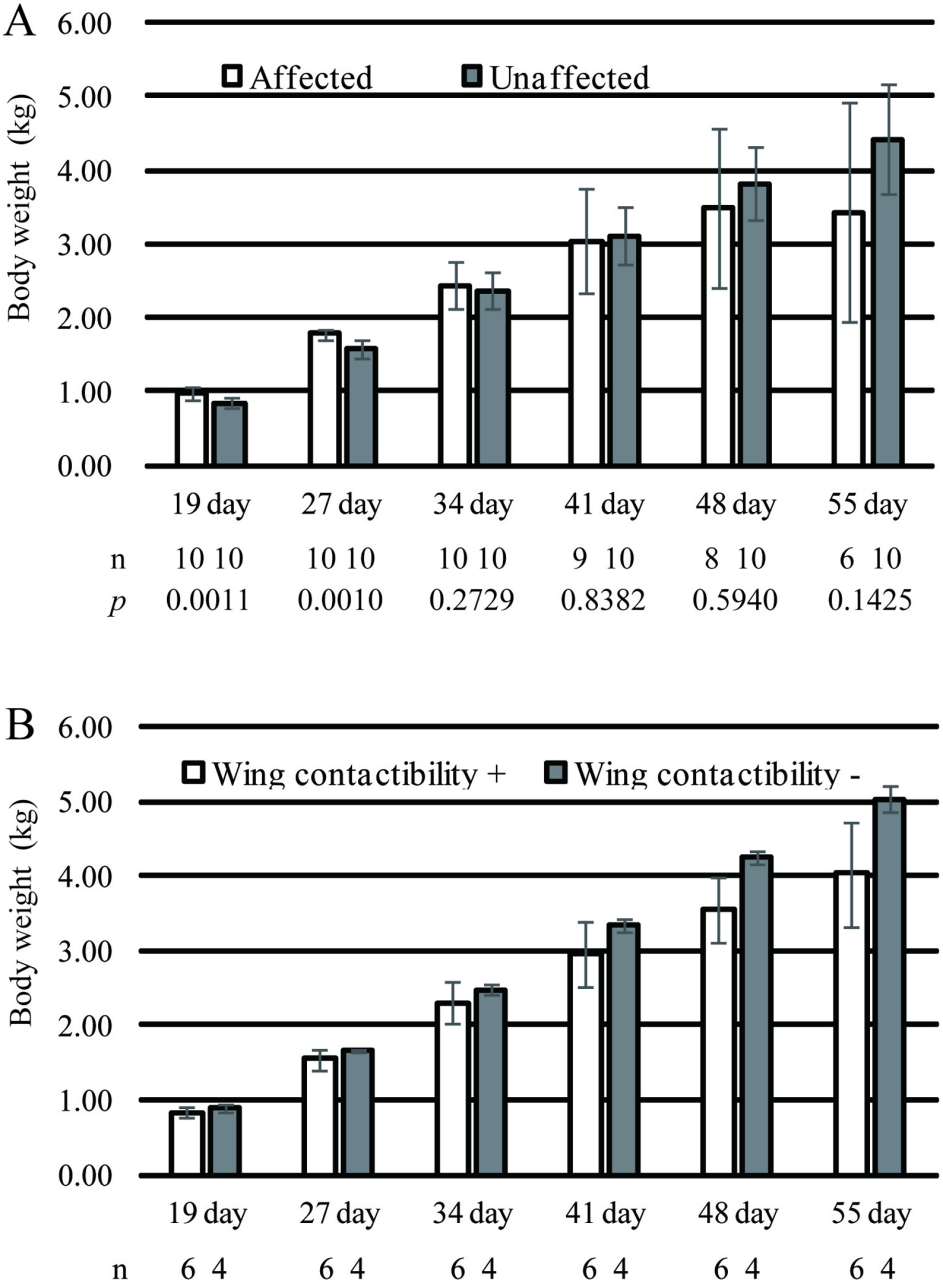
Body weights of affected or unaffected birds during the rearing period (A), and body weights of unaffected birds divided into two groups according to the ability of back-to-back wing contact at 55 days of age (B). The mean body weight gain of affected birds suppressed comparing with that of unaffected birds during the rearing period. Although the number of samples in B is insufficient to obtain statistical significance, the mean body weight of the group without back-to-back wing contact was slightly higher than the mean body weight of the group with back-to-back wing contact through the rearing period.

**Fig 2:**
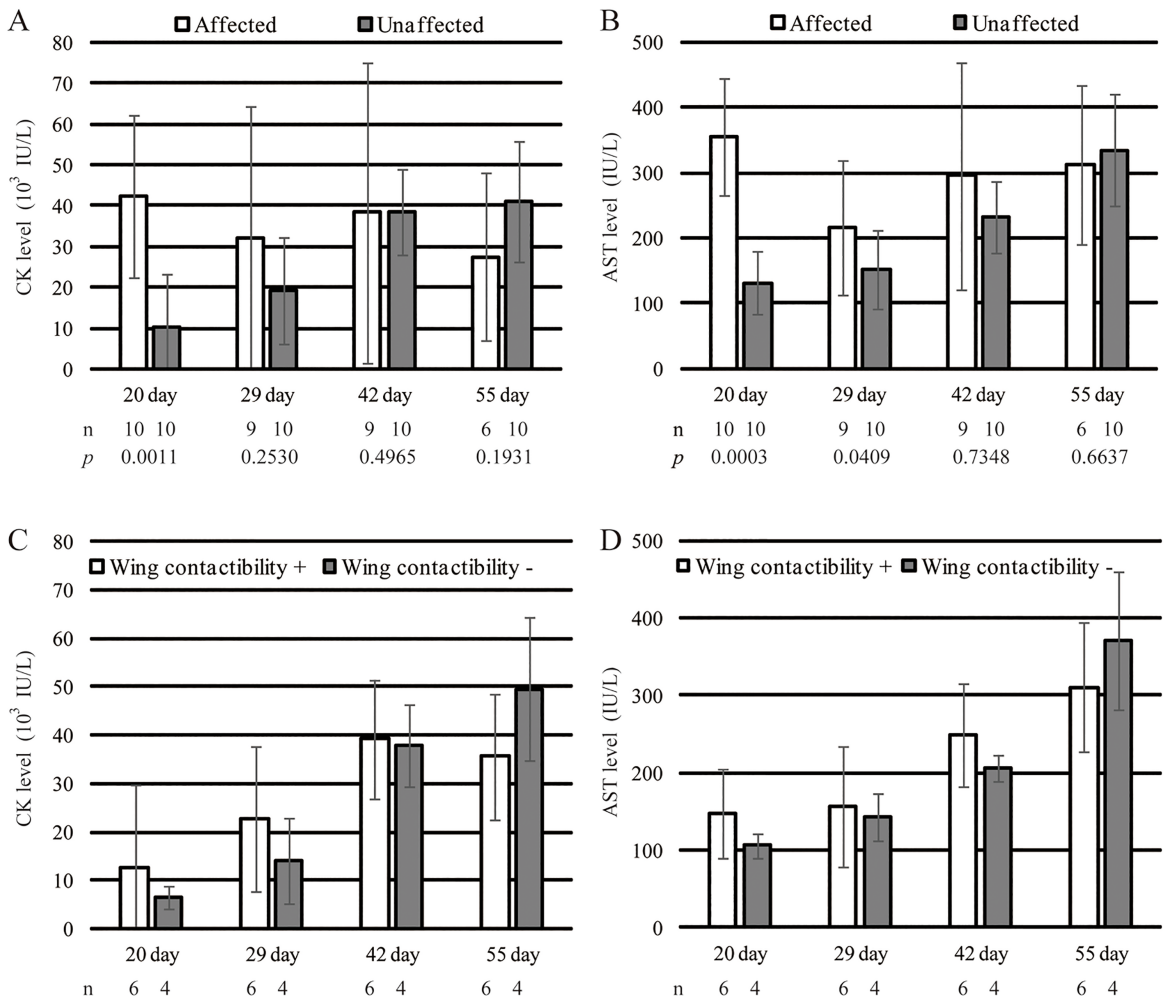
Plasma creatine kinase (CK) and L-aspartate aminotransferase (AST) level examined in 20-, 29-, 42-, and 55-day-old affected and unaffected birds (A, B), and CK and AST levels of unaffected birds divided into two groups according to the ability of back-to-back wing contact at 55 days of age (C, D). The CK and the AST of unaffected birds were increased rapidly than the CK and the AST of affected birds with increasing age. Although the number of samples in C and D is insufficient to obtain statistical significance, CK and AST values of unaffected birds was increased in the group without back-to-back wing contact with increasing age.

The body weights of 19-, 27-, 34-, 41-, 48-, and 55-day-old birds are shown in Fig 1. At 19 and 27 days of age, affected birds were significantly heavier (mean weight, 0.98 ± 0.07 kg) than unaffected birds (mean weight, 0.85 ± 0.07 kg). However, at >41 days of age, the mean weight of affected birds was lower than that of unaffected birds. Among the affected birds, 5 of 10 birds appeared exhausted and exhibited a markedly suppressed weight gain (No. 1, 3, 5, 6, and 7). In addition, 4 of 10 affected birds died (No. 1, 5, 6, and 9) during the rearing period. In contrast, all unaffected birds showed considerable weight gain (No. 19 showed slightly suppressed weight gain during the late stage of rearing) and survived until 55 days of age. Leg weakness due to splayed legs appeared in No. 3, 5, and 7 from 29 to 42 days of age. No. 5 and 6 were affected by ascites. No notable external symptoms were observed among unaffected birds. The ability of the birds to achieve back-to-back wing contact is shown in Table 1. Four of 10 unaffected birds (No. 12, 16, 18, and 20) lost their ability of back-to-back wing contact during late stage of rearing. In contrast, 2 of 6 affected birds (No. 3 and 7) recovered its ability of back-to-back wing contact. In addition, when comparing the unaffected birds divided into two groups according to the ability of back-to-back wing contact at 55 days of age; the mean body weight of the group without achieve back-to-back wing contact was slightly higher than the mean body weight of the group with achieve back-to-back wing contact through the rearing period (Fig 2). Because of the small number of birds, these values were not analyzed with statistically.

**Table 1:** The ability to achieve back-to-back wing contact at 20, 29, 42, and 55 days of age. Four of 10 unaffected birds (No. 12, 16, 18, and 20) lost their ability of back-to-back wing contact during late stage of rearing. In contrast, 2 of 6 affected birds (No. 3 and 7) recovered its ability of back-to-back wing contact.

	Bird No.	Back-to-back wing contact			
		20d	29d	42d	55d
Affected	1	-	-	-	died
	2	-	+	-	-
	3	-	-	+	+
	4	-	-	-	-
	5	-	-	-	died
	6	-	-	died	
	7	-	+	-	+
	8	-	-	-	-
	9	-	-	-	died
	10	-	-	-	-
Unaffected	11	+	+	+	+
	12	+	+	-	-
	13	+	+	+	+
	14	+	+	+	+
	15	+	+	-	+
	16	+	+	-	-
	17	+	+	+	+
	18	+	+	+	-
	19	+	+	-	+
	20	+	+	-	-

### Biochemical analysis

Plasma CK and AST values of affected or unaffected birds at every age are presented in Fig 2. At 20 days of age, the mean plasma CK value in affected birds (42,360 ± 20,077 IU/L) was significantly higher than that in unaffected birds (10,164 ± 13,291 IU/L) (*P* < 0.01). The CK value in No. 19 was 47,760 IU/L as similar level as in affected birds. The mean CK values of unaffected birds showed a steady increase within increasing age. In contrast, the CK values of some affected birds decreased with increasing age. At 55 days of age, the mean CK value in unaffected birds (41,204 ± 14,811 IU/L, n = 10) exceeded that in affected birds (27,540 ± 20,525 IU/L, n = 6) but was not significantly different. In addition, at 20 days of age, the mean plasma AST value in affected birds (356 ± 90 IU/L, n = 10) was significantly higher than that in unaffected birds (131 ± 48 IU/L, n = 10) (*p* < 0.01). The mean AST value in unaffected birds increased with increasing age, and at 55 days of age, the mean AST value in unaffected birds (335 ± 86 IU/L, n = 10) exceeded that in affected birds (312 ± 122 IU/L, n = 6) but was not significantly different. When comparing the unaffected birds divided into two groups according to the ability of back-to-back wing contact at 55 days of age; until 42 days of age, the mean CK and AST values of the group without back-to-back wing contact was somewhat lower than the mean CK and AST values of the group with back-to-back wing contact, however, at 55 days of age, these values of the two groups were reversed (Fig 2). Because of the small number of birds, these values were not analyzed with statistically.

### Postmortem examination

**Fig 3:**
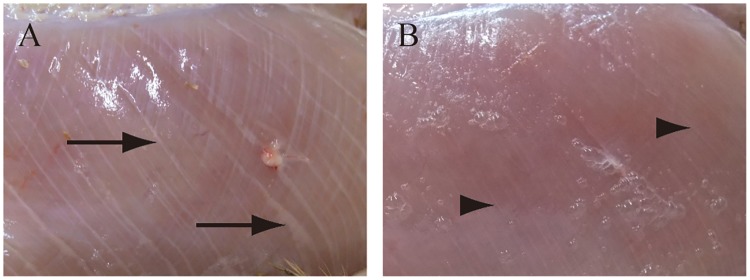
White striping on the surface of PM. Macroscopically, prominent milky or grayish white stripes (arrows) were seen in affected bird No. 2 (A), and slight white stripes (arrow heads) were seen in unaffected bird No. 15 (B).

Two affected birds (No. 3 and 7) were very lean at 55 days of age. The breast-width-per-length ratio of 55-day-old were 0.77, 0.79 and 0.82 mm/mm in affected birds except for two lean birds, 0.52 and 0.57 mm/mm in two affected lean birds, and 0.76 ± 0.02 mm/mm in unaffected birds. The manifestation of white striping on the PM was observed in all birds except No. 3, 7, and 9 (Fig 3). In addition, white striping on SM was observed in No. 1, 5, 6 and No. 12. No inflammatory exudate was observed on the surface of the PM in any bird. Deep pectoral myopathy was observed in the SM of No. 6.

### Histology

With regard to muscle tissue, the mean diameter of myofibers of samples of 55-day-old affected birds observed during postmortem examinations was smaller than that of unaffected birds. The mean diameters of myofibers of samples of 55-day-old in unaffected birds (n = 7) were 48.50 ± 20.18 μm and 51.53 ± 18.50 μm in the PM and SM, respectively. The mean diameters of myofibers in affected birds (No.2, 8 and 10), except for lean birds (No. 3 and 7), were 55.33 ± 24.83 μm, 45.98 ± 20.03 μm and 43.20 ± 20.92 μm in the PM, and were 57.93 ± 19.92 μm, 43.78 ± 17.30 μm and 51.19 ± 16.71 μm in the SM, respectively. The mean diameters of myofibers in the lean birds (No. 3 and 7) were 14.19 ± 4.81 μm and 30.33 ± 13.71 μm in the PM and were 22.69 ± 7.22 μm and 35.73 ± 12.27 μm in the SM, respectively. In addition, the mean diameters of myofibers in the PM and the SM of individual unaffected birds in the two groups divided according to the ability of back-to-back wing contact at 55 days of age showed in Table 2.

**Table 2:** The mean diameters of myofibers in the PM and the SM of individual unaffected birds at 55 days of age. In this study, because the number of birds was small, the mean of muscle fiber diameter and the standard deviation were shown individually. The mean muscle fiber diameter between two groups appears no difference.

Wing contactability	Case No.	PM	SM
+	14	51.01 ± 20.13μm	60.12 ± 16.84μm
	15	48.65 ± 21.38μm	62.73 ± 16.80μm
	17	49.42 ± 19.25μm	42.62 ± 13.25μm
	19	39.79 ± 13.72μm	41.63 ± 12.91μm
-	12	55.96 ± 21.59μm	49.00± 16.71μm
	16	47.83 ± 19.64μm	55.64 ± 18.94μm
	20	46.85± 21.42μm	48.97 ± 21.47μm

**Fig 4:**
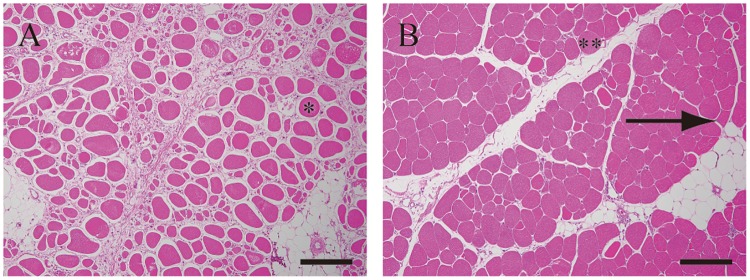
The absence of the characteristic polygonal shape of myofibers in the pectoralis major muscles of affected bird No. 9 (A) and unaffected bird No. 15 (B). Absence of characteristic polygonal shape of myofibers were observed not only in the pectoralis major muscles of affected bird (8 of 9) but also the pectoralis major muscles of unaffected bird (7 of 7). Most of myofibers in affected birds lacked polygonal shape, and appeared round shaped (*) contrasted to polygonal myofibers found in unaffected birds (arrow). However, even in unaffected birds, some myofibers lacked polygons (**). Interstitial thickening with fibrosis and intermuscular adipose tissues observed in affected bird, whereas they are only slightly observed in unaffected bird. The sections were stained with Hematoxylin and eosin stain. Scale bars: 200 μm.

**Fig 5:**
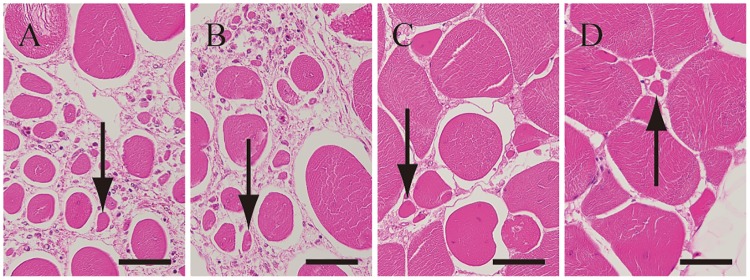
Myofibers of small caliber. Myofibers of small caliber were found not only in the pectoral major muscles but also in the supracoracoideus muscles (9/9 of affected and 7/7 of unaffected), and more in severe lesions with progressive fibrosis. Degenerative changes were more severe in the pectoral major muscles (A) and supracoracoideus muscles (B) of No.9 than the pectoral major muscles (C) and supracoracoideus muscles (D) of No.2. Arrows indicated typical myofibers of small caliber. The sections were stained with Hematoxylin and eosin stain. Scale bars: 50 μm.

The absence of the characteristic polygonal shape of myofibers was observed in most cases in affected and unaffected PM (Fig 4). Further, the same change was observed in most of the SM. Degenerative or necrotic changes in myofibers, increasing intramuscular adipose tissues, and diffuse interstitial thickening were slightly more severe in affected birds than in unaffected birds (Table 3). However, the expression and degree of interstitial thickening with fibrosis showed individual variation. Myofibers of small caliber (< 20 μm) were observed in all examined birds, and the changes were localized or disseminated in the PM or SM or both (Fig 5).

**Table 3:** Assessment of major histological findings of remarkably hardened breast of affected and unaffected birds. Degenerative or necrotic changes in myofibers, increasing intramuscular adipose tissues, and diffuse interstitial thickening were slightly more severe in affected birds than in unaffected birds.

Bird No.	Age at necropsy	Part of muscle	Absence of characteristic polygonal shape on myofiber	Myofiber degenerative changes	Myofiber of small caliber	Intramuscular adipose tissues	Interstitial thickening with fibrosis	Infiltrate of inflammation cells
6	37d	PM	+	+	+	++	+	-
		SM	++	+	+++	-	+	-
5	44d	PM	±	-	+	++	±	-
		SM	+	±	+++	-	-	-
9	52d	PM	+++	++	++	+	++	++
		SM	+++	+++	++	-	+++	++
1	54d	PM	+++	+++	+++	+	++	-
		SM	+	±	+	-	+	-
2	55d	PM	±	+	+	++	±	-
		SM	+	+	+	++	++	+
3	55d	PM	-	-	+++	-	+	-
		SM	+	±	+++	-	-	-
7	55d	PM	±	++	+++	±	+	-
		SM	-	-	+	-	-	-
8	55d	PM	+	+	+	-	±	-
		SM	++	++	++	-	++	-
10	55d	PM	++	++	+	+	+	-
		SM	±	-	±	-	±	-
12	55d	PM	++	+	±	+	+	±
		SM	+	±	+	-	+	-
14	55d	PM	++	+	++	-	++	+
		SM	-	-	±	-	-	-
15	55d	PM	+++	+	+	+	+	-
		SM	-	-	±	+	-	-
16	55d	PM	++	+	+	++	+	-
		SM	+	-	±	-	±	-
17	55d	PM	++	+	+	++	+	-
		SM	±	-	+	±	±	±
19	55d	PM	±	±	++	-	±	-
		SM	-	-	++	-	-	-
20	55d	PM	++	+	+	-	+	-
		SM	±	±	+	+	±	-

-; no findings,

±; slight,

+; mild,

++; moderate,

+++; severe

PM; pectoralis major muscles, SM; supracoracoideus muscles

**Fig 6:**
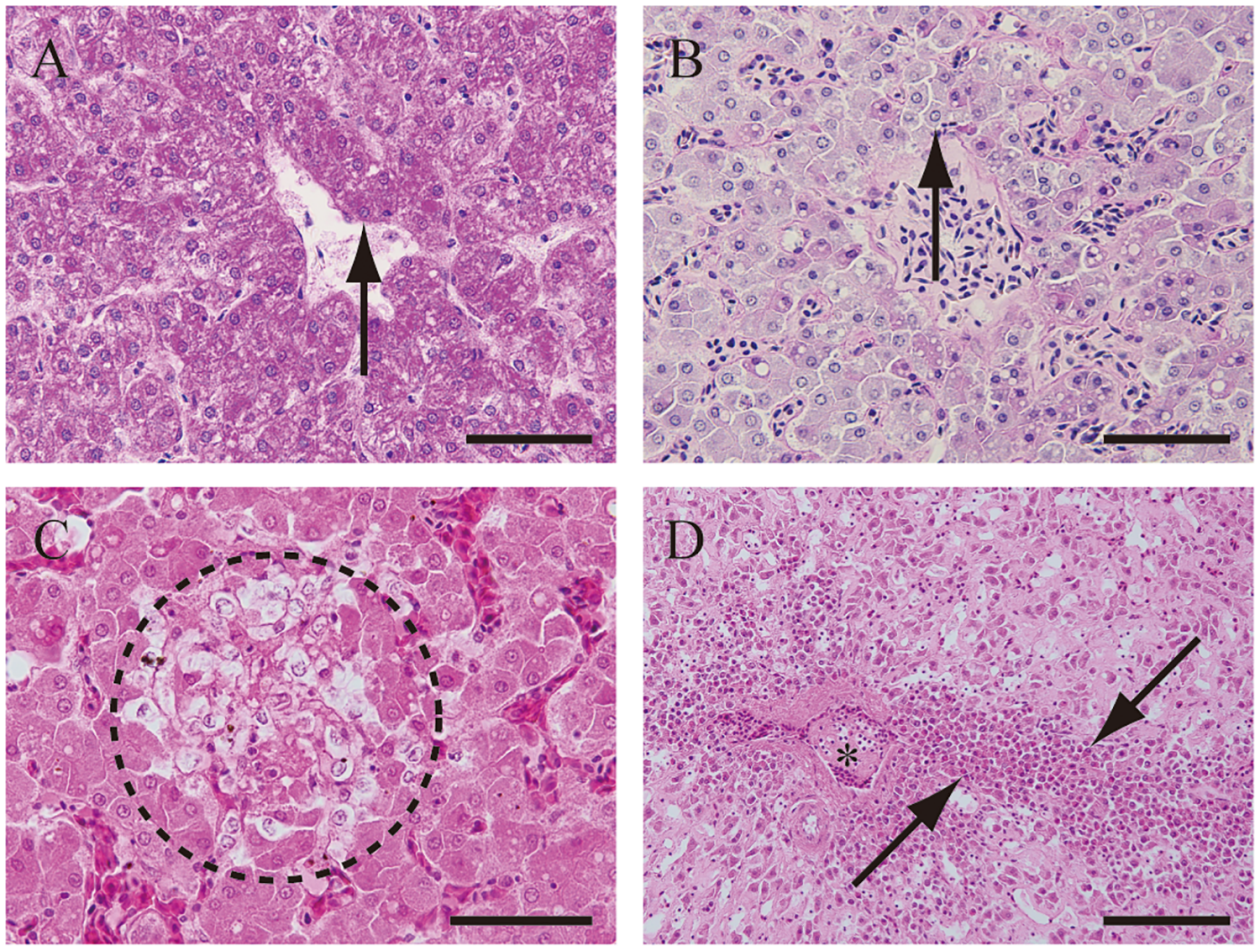
Periodic acid–Schiff reaction properties (A), a necrotic lesion (B) and atrophic changes (C) in hepatocytes. Most of the hepatocytes in the liver of No. 20 were positive with periodic acid–Schiff (A). In contrast, the hepatocytes in the liver of No. 1 stained negative for periodic acid–Schiff (B). Arrows indicate typical hepatocytes respectively. The area enclosed by the dotted circle of C is a micro focal necrotic lesion in the hepatocytes of bird No. 1. Diffuse sinusoidal fibrosis with severe hepatocyte atrophy in the liver of bird No. 3 (D). Fibrin thrombus of blood vessel (*) and perivascular myeloid cell infiltration are observed (arrows). The sections of C, D were stained with Hematoxylin and eosin stain. Scale bar: A-C; 50 μm, D; 100 μm.

Within the liver, most of hepatocytes in 6 of 7 in unaffected birds were periodic acid–Schiff positive, whereas most of hepatocytes in 7 of 9 in affected birds were periodic acid–Schiff negative (Fig 6A and 6B). Focal necrosis in hepatocytes was observed in No. 1, 6, and 8 (Fig 6C). Diffuse sinusoidal fibrosis, severe hepatocyte atrophy, and myeloid cell infiltration were observed in No. 3 (Fig 6D).

**Fig 7:**
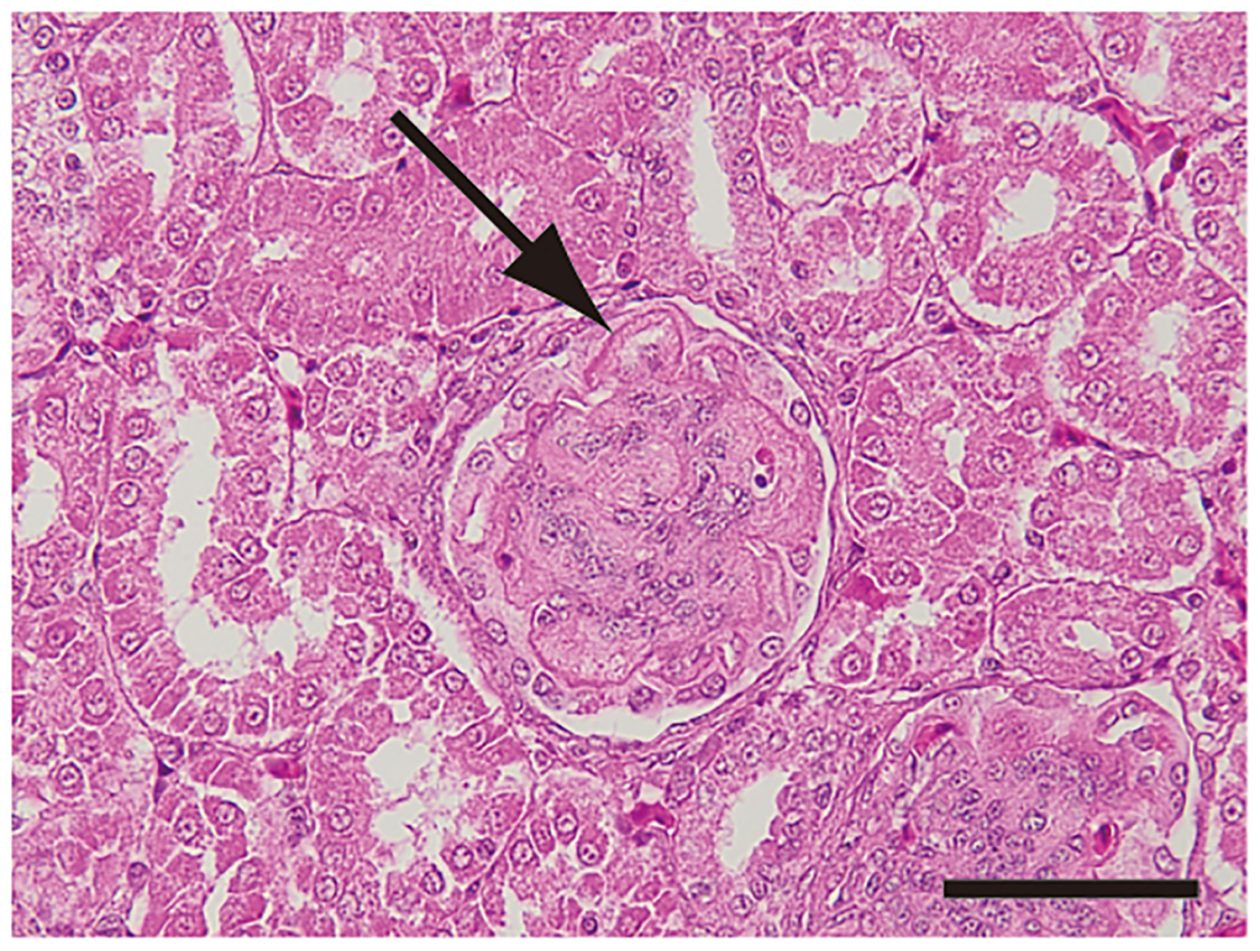
Membranous glomerulopathy. Thickening of glomerular capillary walls (arrow) in the kidney of bird No. 20. The sections were stained with Hematoxylin and eosin stain. Scale bar: 50 μm.

Thickening of glomerular capillary walls without an increase in cellularity (membranous glomerulopathy) was observed in the kidney of No. 1, 3, 8 and No. 14 and 20 (Fig 7).

## Discussion

Damage occurring within the muscle tissue can be reflected in plasma or serum biochemical profiles [6]. CK assay of a blood sample is mainly used as a major marker of skeletal muscle injury. Likewise, because the AST level increases following hepatocellular injury and myocyte injury, it can be considered as a useful marker for evaluating skeletal muscle injury [7]. In the present study, lesions observed in remarkably hardened breast due to damaged PM within selected live birds were diagnosed using a combination of a physical examination and biochemical analysis. CK and AST values in the group of 20-day-old affected birds were higher than those in unaffected group. Therefore, the biochemical profiles indicated that group selection according to the presence of lesions with a physical examination was appropriate.

All unaffected birds had wing contactability until 29 days of age, further 6/10 of these birds kept wing contactability but 4/10 birds lost wing contactability at 55 days of age. CK values in unaffected birds steadily increased with increasing age, indicating that muscular degenerative lesions had been occurring with age in unaffected birds. In contrast, the CK of affected birds decreased with increasing age, reflecting that decreased weight gain suppressed extreme muscle proliferation and reduced the occurrence of muscle degeneration. These relationships between muscle degeneration and changes in CK values were confirmed by findings of absence of polygonal shape or degeneration in muscle fibers of pectoral major muscles at 55 days of age. Also, considering that weight decreasing occurred with a high probability with increasing age in affected birds group, CK and AST values at 20 days of age in unaffected bird of No. 19 might had been reflected the initial changes of muscular degeneration. These observations suggested that the bird was affected with muscular degeneration without apparent physical signs and that elevation in CK and AST values occur before lost wing contactability.

Histologically, similar degenerative lesions of birds affected by remarkably hardened breast were frequently observed not only in the PM but also in the SM. Deep pectoral myopathy is a degenerative lesion of the SM manifested in chickens and turkeys [8]. Shiller et al., [9] described the morphological features of deep pectoral myopathy in the SM of turkeys as focal necrotic lesions found to extend to almost the central region of the muscles, whereas the anterior region of the muscles was usually unaffected. In contrast, in the present study, macroscopic focal necrotic lesions in the central region of the SM were not observed in any cases, except in one bird, and white striping was observed in three affected birds and one unaffected bird. The differentiation suggests that the lesions in most of the SM observed in the present study differed from deep pectoral myopathy. The frequency and severity of muscular lesions of SM were higher in affected birds than that in unaffected birds, and were similar with the lesions of PM.

Peripheral artery disease (PAD) is a manifestation of systemic atherosclerosis that results in progressive narrowing and occlusion of the arteries supplying the lower limb muscles in humans [10,11]. Koutakis et al., [11] described that PAD lesions in the cross sections of muscle tissues were reduced in myofiber size, irregular in shape, included less polygonal and more rounded shapes, and showed decreased myofiber density. Several studies have demonstrated variable diameters of myofibers and an absence of the characteristic polygonal shape in severely degenerated muscles of broilers [1,3,12]. In the present study, the decrease in, or defect of, the characteristic polygonal shape of myofibers was the most common change in the PM not only in affected birds but also in unaffected birds. Myofibers with degenerative changes, small caliber, and interstitial thickening with fibrosis were more commonly seen in affected birds than in unaffected birds. Noteworthy, these muscular findings were also observed in SM. These findings suggested that decrease or defect in the characteristic polygonal shape of myofibers were degenerative findings of primary stages due to chronic insufficient supply of blood, also unaffected birds had the risk of developing the symptoms with increasing age. Moreover, further studies concerning the progressive narrowing and occlusion of the arteries supplying in breast muscles are needed to reveal the degenerative mechanism of remarkably hardened breast and wooden breast. In addition, despite supplying well controlled environment and suitable commercial broiler feed, most affected birds died or developed a serious health condition during the rearing period, and 40–50% of unaffected birds lost their ability of back-to-back wing contact during the late stage of rearing. The results suggested that the extreme development of the breast up to the middle stage of rearing damaged the breast muscles and adversely affected productivity. In this study, since there were only 7 unaffected birds subjected to the tissue examination, the association between the loss of wing contactability expressed in unaffected birds during the rearing period and the diameter and variation of muscle fibers could not be compared statistically.

Within the liver, periodic acid–Schiff positive hepatocytes were more frequently observed in unaffected birds, which suggested that metabolic condition or nutritional absorption concerning glycogenolysis or glycogenesis differed in unaffected and affected birds. Regarding the glycogen content, Abasht et al., [13] have reported that glycogen content measurements in the PM taken in wooden breast-affected birds were considerably lower than those in unaffected birds. Hepatic glycogen synthase deficiencies lead to decreased glycogen stores in mammals [14]. In the disorder, only glycogen synthesis in the liver is impaired, causing postprandial hyperglycemia after ingestion of a carbohydrate-containing meal [15]. In the present study, the possibility of hepatic glycogen synthase reduction in the affected birds was not mentioned. Focal necrosis in hepatocytes was observed in 3 affected birds; severe hepatocyte atrophy was observed in the liver of a very lean bird. These liver changes were presumed to reflect functional deterioration and weakness in affected birds. However, only the findings in this study could not reveal the relationship between the histologic changes in the breast muscles and in the liver. Further studies including biochemical analysis from a multilateral perspective as well as measurement of blood glucose and enzymatic analysis of the liver are required.

The most frequent findings in the kidney were membranous glomerulopathies, which were found in 3 affected and 2 unaffected birds. The lesion is induced by chronic exposure with foreign proteins, resulting in the formation of circulating immune complexes that deposit in the glomerular membranes [16]. The direct cause effect relationship of membranous glomerulopathy does not always exist between the primary disease and renal lesion, but may result from dehydration. Also, it should be considered that the relation with excess sodium chloride in the diet, systemic hypertension, administration of desoxycorticosterone acetate, and aflatoxicosis [17].

We could not get enough data to clarify the definitive mechanism of symptoms in the present study. However, we revealed changes in individual chicken condition over time. As a result, we could obtain some findings that might to be related to the development of the hardened breast.

## Conclusions

In this report, we demonstrated that most broilers having remarkably hardened breast affected during the early stage of rearing would have suppressed physical status and weight gain or would cause death. Moreover, it was suggested that remarkably hardened breast may be a result of rapid growth in broilers.

## Supporting information

S1 File NC3Rs ARRIVE guidelines checklist. (PDF)
